# Medicine as Practiced by Animals

**Published:** 1883-09

**Authors:** 


### SELECTIONS.

### Medicine as Practiced by Animals.

The following article from the *British Medical Journal* is interesting and instructive, proving, as it does, how superior the instinct of animals is to medical science. It deserves to be read carefully, sentence by sentence:—

M. G. Delaunay, in a recent communication to the Biological Society, observed that medicine, as practiced by animals, is thoroughly empirical, but that the same may be said of that practiced by inferior human races, or, in other words, by the majority of the human species. Animals instinctively choose such food as is best suited to them. M. Delaunay maintains that the human race also shows this instinct, and blames medical men for not paying sufficient respect to the likes and dislikes of the patients, which he believes to be a guide that may be depended on. Women are more often hungry than men, and they do not like the same kinds of food; nevertheless, in asylums for aged poor, men and women are put on precisely the same regimen. Infants scarcely weaned are given a diet suitable to adults, meat and wine, which they dislike and which disagree with them. M. Delaunay investigated this question in the different asylums of Paris, and ascertained that children do not like meat before they are about five years of age. People who like salt, vinegar, etc., ought to be allowed to satisfy their tastes. Lorain always taught that, with regard to food, people's likings are the best guide. A large number of animals wash themselves and bathe, as elephants, stags, birds, and ants. M. Delaunay lays down as a general rule that there is not any species of animal which voluntarily runs the risk of inhaling emanations arising from their own excrement. Some animals defecate far from their habitations; others bury their excrements; others carry to a distance the excrement of their young. In this respect they show more foresight than man, who retains for years excrement in stationary cesspools, thus originating epidemics. If we turn our attention to the question of reproduction, we shall see that all mammals suckle their young, keep them clean, wean them at the proper time, and educate them; but these maternal instincts are frequently rudimentary in women of civilized nations. In fact, man may take a lesson in hygiene from the lower animals. Animals get rid of their parasites by using dust, mud, clay, etc. Those suffering from fever restrict their diet, keep quiet, seek darkness and airy places, drink water, and sometimes even plunge into it. When a dog has lost its appetite it eats that species of grass known as dog's grass (*chiendent*), which acts as an emetic and purgative. Cats also eat grass. Sheep and cows, when ill, seek out certain herbs. When dogs are constipated they eat fatty substances, such as oil and butter, with avidity, until they are purged. The same thing is observed in horses. An animal suffering from chronic rheumatism always keeps as far as possible in the sun. The warrior ants have regularly organized ambulances. Latreille cut the antennæ of an ant, and other ants came and covered the wounded part with a transparent fluid secreted from their mouths. If a chimpanzee be wounded, it stops the bleeding by placing its hand on the wound, or dressing it with leaves and grass. When an animal has a wounded leg or an arm hanging on, it completes the amputation by means of its teeth. A dog on being stung in the muzzle by a viper was observed to plunge its head repeatedly for several days into running water. This animal eventually recovered. A sporting dog was run over by a carriage. During three weeks in winter it remained lying in a brook, where its food was taken to it; the animal recovered. A terrier dog hurt its right eye; it remained lying under a counter avoiding light and heat, although habitually it kept close to the fire. It adopted a general treatment, rest, and abstinence from food. The local treatment consisted in licking the upper surface of the paw, which it applied to the wounded eye, again licking the paw when it became dry. Cats also, when hurt, treat themselves by this simple method of continuous irrigation. M. Delaunay cites the case of a cat which remained for some time lying on the bank of a river; also that of another cat which had the singular fortitude to remain for forty-eight hours under a jet of cold water. Animals suffering from traumatic fever treat themselves by the continued application of cold, which M. Delaunay considers to be more certain than any of the other methods. In view of these interesting facts, we are, he thinks, forced to admit that hygiene and therapeutics, as practiced by animals, may, in the interests of psychology, be studied with advantage. He could go even further, and say that veterinary medicine, and perhaps human medicine, could gather from them some useful indications, precisely because they are prompted by instinct, which are efficacious in the preservation or the restoration of health.

"Go to the ant, thou sluggard; consider her ways and be wise." The most admirable inventions of men are, in a multitude of instances, imitations of nature. If we ever navigate the air it will be by machines constructed from observation of birds or insects. We find some good observations on the above in a leader in the *Glasgow Herald.* The writer says:—

"Many species of animals wash themeslves with regularity, and at every available opportunity. Birds are much given to ablution, and an elephant takes its bath with undeviating delight. The deer also bathe; and it is said that those eminently 'social' insects, the ants, also emulate in this respect the example of higher life. Nor is this apparent desire for cleanliness the only characteristic of animal life. It may be that the bath in which animals indulge is only a form of luxury; but even on this view of matters the animal shows to advantage as compared with many civilized people, who regard bathing neither as a luxury nor as a necessity. The extreme care with which most animals rid themselves of annoying insect or parasitic visitors is also remarkable. The monkeys, contrary to popular belief, are very cleanly animals. Their habit of cleaning their fur is one which precludes the idea of insect pests being allowed to infest their hair. Those who have studied the monkey tribes assure us that no parasites exist on the skins of these animals, and that they are more than careful of their fur. The elephant rids itself of its insect pests, and protects its skin from further attack by the mud-bath in which it delights; and many other quadrupeds similarly contrive to avoid the annoyance of insect visitors.

"In the treatment of actual disease, animals exhibit many interesting features. What is to be said of those belligerent ants that have organized an 'ambulance corps' to deal with the maimed and the hurt? Or how can we explain on other than rational grounds the fact that when a wounded ant had been bereft of its 'feelers,' its neighbors came to the rescue, and soothed the wounded insect by covering the injured part with the secretion of their mouths? This fact is, after all, not more wonderful than many other phases of ant existence. It cannot be surprising to find attention paid to the wounded by insects which have succeeded in domesticating the young of other species of ants as slaves, and which keep plant lice as men keep cows, for the sake of the sweet secretion the plant lice afford. Sir John Lubbock has an interesting paragraph or two on the subject of the treatment of drunken ants by their sober and rational neighbors. Out of 41 intoxicated ant friends, 32 were carried off to the nest, and nine were thrown into water, this last a most effective medical antidote —the cold douche—for inebriety. Out of 52 drunken ants, which were strangers to the particular nest, near which they were placed, only two were taken into the nest whilst 50 were thrown into the water. Even the two that were housed, Sir John Lubbock believes, may have been subsequently douched. There seems every reason to believe that the ants thus indulged in a highly legitimate form of medical practice. The cold water cure has been long esteemed as a remedy for the foolishness that results in a debauch. But it would seem that many other animals utilize cold as an antidote to, or remedy for, fevers and inflammations. Some natural instinct akin to that which guides savage mankind in their choice of remedies appears to direct the medical and surgical efforts of animals. A fevered animal restricts its diet, it is true; but this result may accrue from loss of appetite. Then water is copiously utilized in the treatment. Inflamed parts are regularly bathed in cold water, and animals have been known to exert themselves in a literally amazing degree to bring their inflamed parts within reach of the cooling application. In the treatment of a wounded and inflamed eye, a monkey has been known to apply cold water to the injured organ almost constantly."

We knew of the case of a horse wounded in the neck, which held the wound several times a day under a small douche of water. The most clever hydropathist could not have prescribed better treatment. Yet our philosophers tell us that there is no such thing as instinct. In that case animals must have experimented and thought out all these modes of medical treatment, and all the wonderful things they do. Spiders invented their webs and the apparatus with which they make the material and spin it. Bees invented their combs, and the mode of making wax, honey, the poison of their stings, and the glands to secrete it, and the instrument which makes it effective.—*London Herald of Health.*

